# Dynamic oxygenator blood volume during prolonged extracorporeal life support

**DOI:** 10.1371/journal.pone.0263360

**Published:** 2022-02-02

**Authors:** Rik H. J. Hendrix, Eva R. Kurniawati, Sanne F. C. Schins, Jos G. Maessen, Patrick W. Weerwind

**Affiliations:** Department of Cardiothoracic Surgery, Maastricht University Medical Centre, Maastricht, The Netherlands; Monash University, AUSTRALIA

## Abstract

Current methods for identification of oxygenator clotting during prolonged extracorporeal life support include visual inspection, evaluation of oxygenator resistance and oxygen exchange performance, and assessment of clotting-related laboratory parameters. However, these observations do not provide a quantitative assessment of oxygenator clot formation. By measuring changes in the dynamic oxygenator blood volume this study aimed to evaluate the relation to oxygenator resistance and oxygen transfer performance. Sixty-seven oxygenators were studied during adult extracorporeal life support. Oxygenator blood volume, oxygenator resistance, and oxygen transfer efficiency were monitored. Oxygenator blood volume decreased with increasing runtime (r = -0.462; p <0.001). There was a statistically significant, fair negative correlation between oxygenator blood volume and oxygenator resistance (r = -0.476; p<0.001) in all oxygenators, which became stronger analyzing only exchanged oxygenators (r = -0.680; p<0.001) and oxygenators with an oxygenator blood volume <187 mL (r = 0.831; p<0.001). No relevant correlation between oxygenator blood volume and O_2_ transfer was found. Oxygenator blood volume declined over time and was clearly associated with an increasing oxygenator resistance during prolonged extracorporeal life support, though O_2_ transfer was less affected.

## Introduction

In the last two decades, extracorporeal life support (ECLS) technology has greatly improved, but the blood-biomaterial interaction with inherent systemic responses remains an unsolved problem with frequent and often unpredictable system occurrences. Blood contact, even with the biocompatible-coated material of the ECLS circuit leads to an immediate stimulation of the coagulation cascade [1–3]. Along with the properties of the artificial circuitry, shear forces and turbulence associated with blood flow rates generated during ECLS add to this activation [1]. Systemic anticoagulation is applied in an attempt to counteract the prothrombotic effect of the ECLS circuit, but even in adequately anticoagulated patients, clot formation in circuit components occurs. The component most vulnerable for clot development is the oxygenator, which has a large foreign surface area and instigates high shear forces. This increases the risk for platelet deposition on the membrane fibers and further augmentation of the procoagulant state, ultimately increasing the clot formation potential. Intra-oxygenator clot formation can lead to decreased oxygen transfer by increasing the diffusion distances of gasses when forming deposits on the membrane fibers, or by blocking blood flow to certain membrane regions [4]. Clots will further exacerbate the coagulation process and lead to an increased risk for thromboembolic complications when located on the arterial side of the oxygenator. These complications, or merely the risk thereof, can necessitate oxygenator replacement, but the right moment to replace an oxygenator is difficult to determine in daily practice, as all changeouts pose a certain risk to the patient and unnecessary circuit changes should thus be avoided.

Current methods of monitoring intra-oxygenator clot formation include visual inspection of the oxygenator, measuring oxygenator resistance, analyzing the gas exchange performance, and assessment of laboratory parameters (e.g. free hemoglobin, D-dimers) [5–7]. All these methods have their own disadvantages. Visual inspection only reveals clot formation on the visual part of the oxygenator membrane. An increase in oxygenator resistance due to mechanical membrane blockage by clots depends not only on the size of the clot, but also on its location in the oxygenator. Clots in low flow areas of an oxygenator will cause less resistance than clots in high flow areas [8]. Consequently, relative changes in oxygenator resistance are not proportional to the amount of clot formation [8, 9]. Oxygen transfer performance can also be decreased by fluid accumulation in the gas compartment [10–12]. Increased D-dimer levels are linked to varying underlying conditions (e.g. disseminated intravascular coagulation, major surgery), whereas increased levels of plasma free hemoglobin during ECLS are often described as a resultant of blood product usage or high pump rotations (i.e. mechanical blood damage), which are all quite common in ECLS patients [13–15]. Thus, there are currently no reliable techniques for direct visualization and accurate quantification of imminent clot formation inside the oxygenator.

An in vitro validation study of the extracorporeal life support assurance (ELSA) monitor (Transonic systems, Ithaca, USA) showed that a decrease in oxygenator blood volume (OXBV, i.e., the effectively perfused volume of the oxygenator) is associated with intra-oxygenator clot development [8]. In this study, changes in OXBV were evaluated in a clinical setting during prolonged ECLS in relation to oxygenator resistance and oxygen transfer efficiency.

## Materials and methods

### Patients

This observational clinical study included adult patients supported with either veno-venous (VV) or veno-arterial (VA) ECLS for three or more consecutive days between March 2018 and January 2020. A Bioline-coated Permanent Life Support (PLS) system including a polymethylpentene oxygenator (PLS-i) and a Rotaflow centrifugal pump (Getinge, Gothenburg, Sweden) was used in all cases. Post-cardiotomy patients did not receive heparin in the first 24 hours after surgery. All other patients received a bolus of unfractionated heparin (100 IU/kg) before cannulation, and a continued heparin infusion aiming at an activated partial thromboplastin time (aPTT) of 50–60 seconds. Heparin infusion was temporarily stopped in case of substantial bleeding.

Data acquisition and analysis in this observational study were performed anonymously and in accordance with the Dutch law for approving medical research. The Clinical Trial Center Maastricht approved this study and stated that it is not a study covered by the law on medical research involving human subjects (clinical trial center registration number METC 2020–2110). Since the study didn’t influence the routine care of the patient, informed consent was waived.

### Measurements and calculations

The ELSA monitor (Transonic systems) was used to measure and calculate OXBV. A saline infusion system consisting of a sampling manifold line, a bag of saline and a syringe were connected to a pre-oxygenator luer lock. The arterial ELSA sensor was placed at the arterial outlet of the oxygenator and the venous sensor was placed on the venous line as close to the patient as possible. The working mechanism of the ELSA monitor and the calculation of the OXBV are described elsewhere [8]. The OXBV was, when possible, measured in twofold daily for as long as the patient received extracorporeal support. The trans-oxygenator pressure gradient (Δp), measured via Truwave pressure monitoring sets (Edwards Lifesciences Corporation, Irvine, CA, USA), was used to calculate oxygenator resistance:

Resistance(mmHg/L/min)=ΔpQp (1)

Where Q_p_ is the pump flow. Pre- and post-oxygenator blood gas samples were analyzed using the GEM premier 4000 blood gas analyzer (Instrumentation Laboratory, Bedford, MA, USA). Post-oxygenator and pre-oxygenator O_2_ content (CaO_2_ and CvO_2_ respectively) were calculated in order to determine O_2_ transfer:

$${\mathrm{CaO}}_{2}\;(\mathrm{mL}/\mathrm{dL})=(\mathrm{Hb}\times 1.36\times {\mathrm{SaO}}_{2})+({\mathrm{PaO}}_{2}\times 0.003)$$ (2)

$${\mathrm{CvO}}_{2}\;(\mathrm{mL}/\mathrm{dL})=(\mathrm{Hb}\times 1.36\times {\mathrm{SvO}}_{2})+({\mathrm{PvO}}_{2}\times 0.003)$$ (3)

$$O_{2}\;\mathrm{transfer}\;(\mathrm{mL}/\mathrm{dL})={\mathrm{CaO}}_{2}‐{\mathrm{CvO}}_{2}$$ (4)

Where Hb is the hemoglobin concentration in g/dL, 1.36 is a constant (mL O_2_/gr hemoglobin), SaO_2_ and SvO_2_ are the post- and pre-oxygenator O_2_ saturation (%), PaO_2_ and PvO_2_ are the post- and pre-oxygenator oxygen tension (mmHg) respectively, and 0.003 is a constant (mL O_2_/mmHg).

Additionally, the oxygen gradient and oxygen diffusing capacity were calculated in order to evaluate oxygenator performance:

$$O_{2}\;\mathrm{gradient}\;(\mathrm{kPa})={\mathrm{pgO}}_{2}‐{\mathrm{paO}}_{2}$$ (5)

O2diffusingcapacity(mL/min/kPa)=O2transferpgO2‐pvO2 (6)

Where pgO_2_, paO_2_ and pvO_2_ are the partial O_2_ pressures in the sweep gas, the post-oxygenator blood and the pre-oxygenator blood respectively. In order to analyse oxygenator performance at the oxygenator’s maximal capacity [16], these analyses were performed only on data with a blood flow rate of at least 3 L/min, a gas flow rate of at least 2 L/min and at 100% FiO_2_. A decrease in post-oxygenator pO_2_ >50% at the same or increased blood and gas flow rates was defined as worsened O_2_ exchange capacity according to Lubnow [6], as was a post-oxygenator pO_2_ <200mmHg [16].

### Statistical analysis

Statistical analysis was performed using SPSS version 25 (IBM Corp., Chicago, IL, USA). Means were compared using independent samples t-tests and Spearman correlation analysis was used to explore correlations. A p<0.05 was considered statistically significant.

## Results

Fifty-two patients supported with ECLS were included in this study (Table 1). In 29 cases VA-ECLS was initiated, whereas 23 patients received VV-ECLS support. During the VA-ECLS runs two patients required a single oxygenator changeout (7%), while one patient required two oxygenator changeouts (3%). In the VV-ECLS cohort four patients required a single oxygenator exchange (17%), while three patients needed two oxygenator exchanges (13%). One patient was initially weaned from VV-ECLS support, but required ECLS again within a week. This resulted in data of 67 oxygenators, 14 thereof requiring replacement (21%).

**Table 1:** Patient demographics.

Male (n (%))		36 (71)
Age (years)		58 ± 11
Height (cm)		175 ± 7
Weight (kg)		85 ± 21
Support mode	VA-ECLS	57%
	VV-ECLS	43%

VA-ECLS—veno-arterial extracorporeal life support; VV-ECLS—veno-venous extracorporeal life support.

The median runtime of exchanged oxygenators was 10.5 [5–13.3] days, compared to 7 [5–11] days in oxygenators not requiring changeout. The aPTT was 58 ± 20 seconds in oxygenators that did not require exchange and 59 ± 24 seconds in those that were exchanged (p = 0.832). Oxygenator resistance was 5.0 ± 1.1 mmHg/L/min at ECLS initiation. In non-exchanged oxygenators the resistance was 6.0 ± 2.4 mmHg/L/min at the last measured day, whereas exchanged oxygenators had a resistance of 13.8 ± 14.5 mmHg/L/min by then.

**Fig 1:**
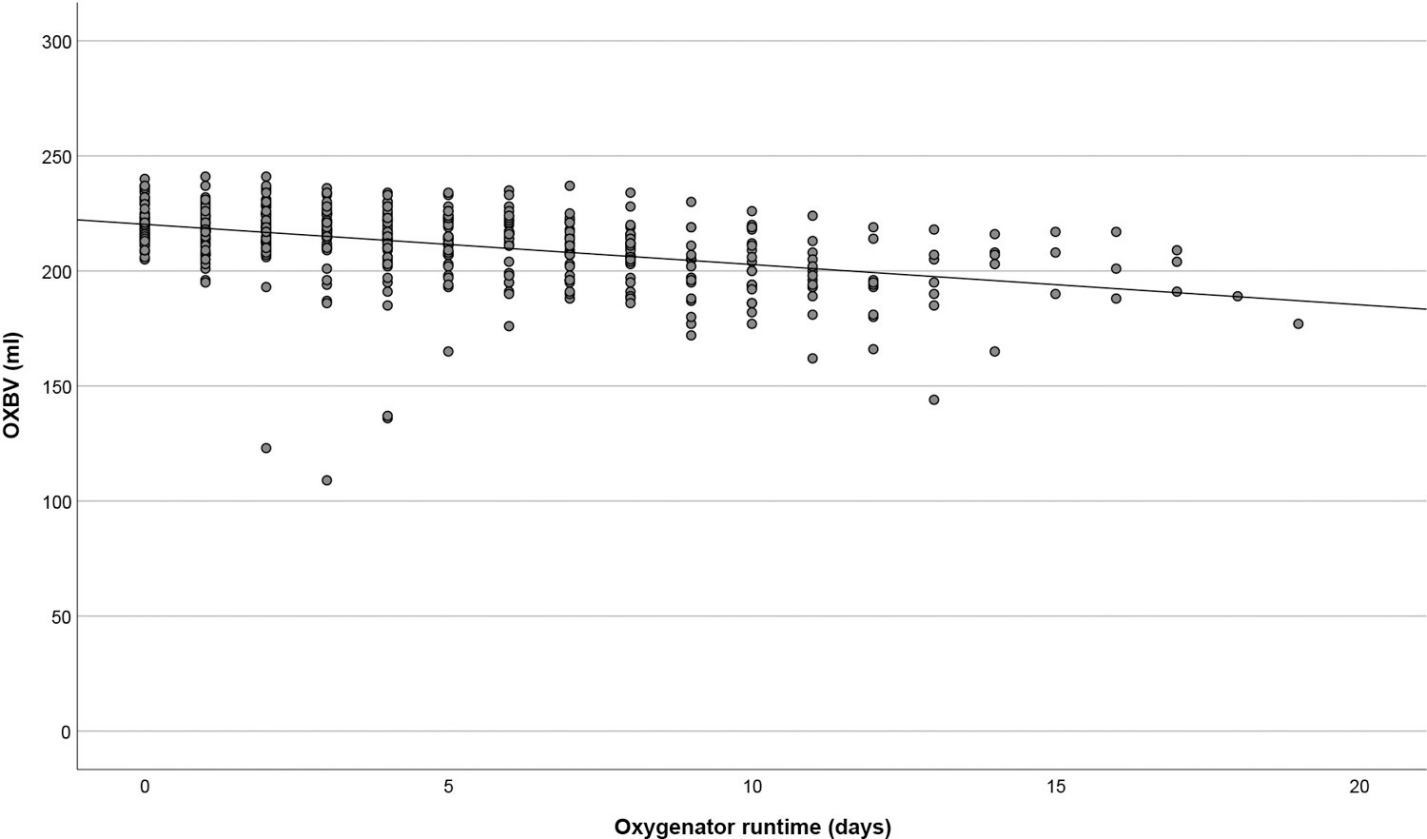
Oxygenator blood volume during the extracorporeal life support runs. OXBV = oxygenator blood volume.

The baseline OXBV was 220 ± 9 mL and decreased during the course of extracorporeal support (Fig 1). Correlation analysis revealed a fair negative correlation between the OXBV and oxygenator runtime (r = -0.462; p<0.001). A decrease from the initial OXBV between zero and five percent was measured in 28 oxygenators during their run, a 6–10% decrease in 15 oxygenators, a 11–20% decrease in 16 oxygenators and a 21–30% decrease in three oxygenators. Five oxygenators showed a decrease in OXBV of more than 30% (34, 36, 37, 46, and 54% decrease, respectively), all with a sudden drop after an OXBV>200 mL the previous day.

Changes in OXBV showed not to be affected by pump flow rate (r = -0.027; p = 0.558). On the other hand, a fair negative correlation between OXBV and oxygenator resistance (r = -0.476; p<0.001) was found.

**Fig 2:**
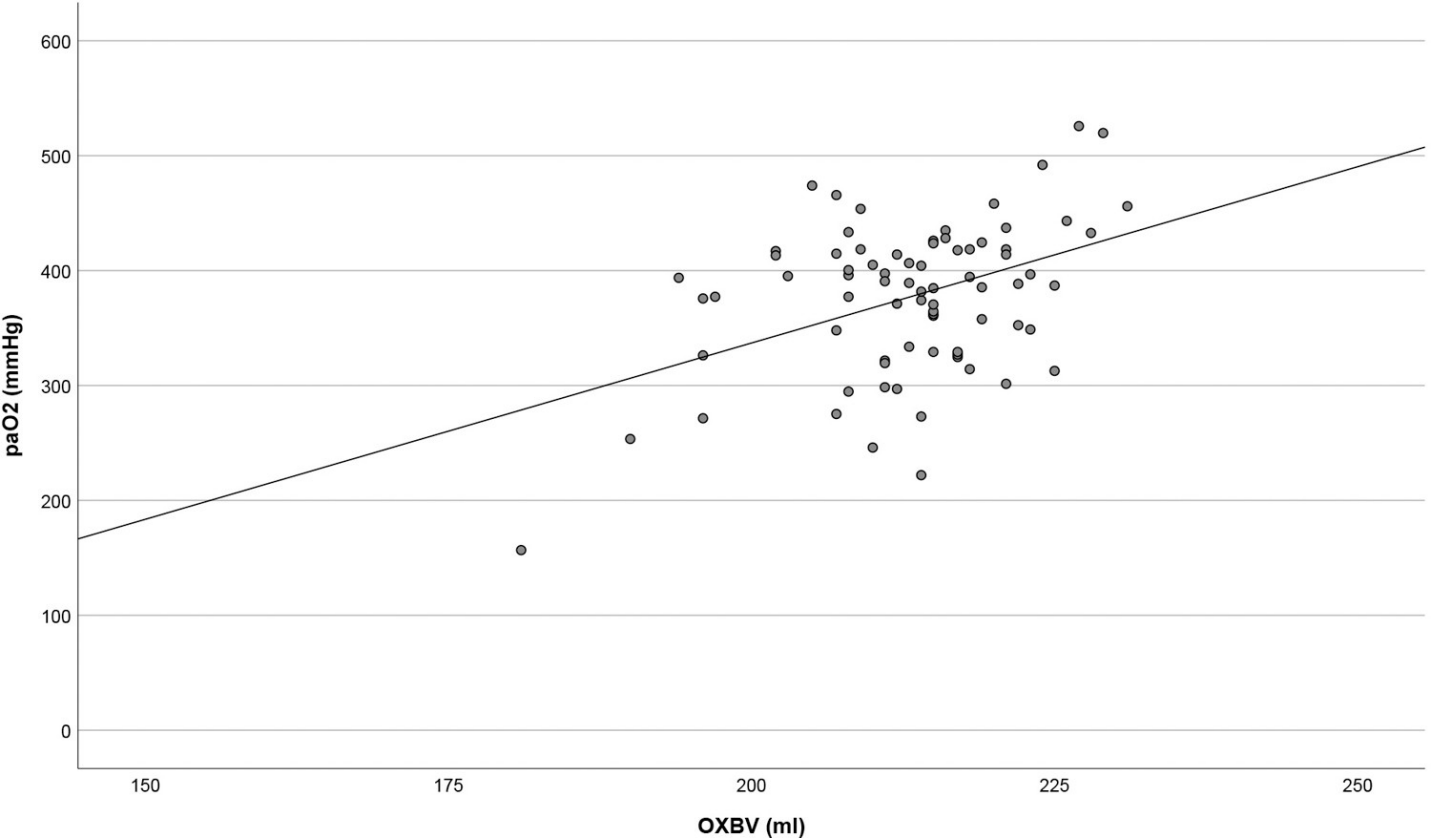
Average OXBV vs. post-oxygenator pO_2_. Represented is all data with a minimum blood flow rate of 3 L/min, a minimum sweep gas flow of 2 L/min and a fiO_2_ of 100%. paO_2_ = post-oxygenator pO_2_, OXBV = oxygenator blood volume.

Analysis of the oxygen transfer efficiency revealed no relevant correlations between OXBV and O_2_ transfer (r = 0.216; p = 0.058) and O_2_ diffusing capacity (r = 0.217; p = 0.056). OXBV and O_2_ gradient showed a fair negative correlation (r = -0.461; p<0.001). A scatterplot of the average OXBV and corresponding post-oxygenator pO_2_ illustrates a fair correlation (r = 0.423; p<0.001), indicating a trend towards a decreased post-oxygenator pO_2_ with decreasing OXBV (Fig 2).

In the patients with the oxygenator functioning at maximal capacity a >50% decrease in post-oxygenator pO_2_ and a post-oxygenator pO_2_<200 mmHg were found in two cases. In the first case the initial pO_2_ of 437 mmHg decreased to 157 mmHg and in the second case the initial pO_2_ of 423 mmHg decreased to 192 mmHg.

The average OXBV at the time of exchange was 167 ± 31 mL. Data from these oxygenators only revealed a moderate negative correlation between OXBV and resistance (r = -0.680; p<0.001), and OXBV and oxygenator runtime (r = -0.642; p<0.001). Correlations between OXBV and O_2_ transfer indices were all weak (OXBV–O_2_ transfer r = 0.256; p = 0.006; OXBV–O_2_ gradient r = -0.219; p<0.018; OXBV–O_2_ diffusing capacity r = 0.137; p = 0.144).

In 27 measurements OXBV declined to 187 mL or less during the course of ECLS, a >15% decrease in the effectively perfused oxygenator volume. When selecting only these data, a strong negative correlation between OXBV and oxygenator resistance (r = -0.831; p<0.001) was observed. Further, no significant correlations were found between OXBV and oxygenator runtime (r = 0.368; p = 0.059), post-oxygenator pO_2_ (r = -0.056; p = 0.800), O_2_ transfer (r = -0.104; p = 0.620), O_2_ gradient (r = -0.231; p = 0.247) and O_2_ diffusing capacity (r = 0.233; p = 0.263).

## Discussion

Clot formation in the ECLS circuit can require an emergent system changeout with inherent risks for the patient. Timely recognition of system clotting gives the possibility to plan an elective, controlled system exchange at a much lower patient risk. Every exchange though, still has its risks, and thus an unnecessary exchange of a well-functioning system should be avoided.

In the current study 14 out of 67 oxygenators (21%) were changed out, after a runtime of 10.5 [5–13] days, which is in a similar range as the PLS-i life span of 8 [7–12] days described by Philipp et al. [17].

The aPTT of the exchanged oxygenators (59 ± 24 seconds) showed a large variability and the low level of anticoagulation in some of these oxygenators could have contributed to enhanced clot development. The non-exchanged oxygenators however, had a similar aPTT (58 ± 20), and all oxygenators exhibited at least some degree of visible clot formation. A similar observation is described in a study by Dornia et al., who analyzed oxygenators after successful weaning using multi-detector computed tomography and found thrombotic deposits in all analyzed oxygenators [18]. The higher odds of oxygenator clotting during the course of VV-ECLS might be due to the longer support time compared to VA-ECLS (310 ± 247 hours vs. 200 ± 129 hours, p = 0.042). Moreover, VV-ECLS cases had more extended use of a single oxygenator compared to VA-ECLS cases (219 ± 103 hours vs. 166 ± 83 hours, p = 0.026). Thus the time for intra-oxygenator clots to develop was prolonged in VV-ECLS.

The average OXBV at baseline was 220 ± 9 mL, which is slightly higher than the actual priming volume of 215 mL for the oxygenator used. This discrepancy can be attributed to the additional volume of tubing between the injection port and the arterial ELSA probe that was not corrected for. Similar to an earlier in vitro study using low blood flow rates [8], the current clinical study found no correlation between OXBV changes and high pump flow rates (r = -0.027; p = 0.558). The decrease in OXBV during the course of extracorporeal support showed to be more prominent in the 14 oxygenators that required replacement (r = -0.642; p <0.001). This is expected, as due to the longer runtime of these oxygenators more clots developed and OXBV had a larger drop. During most cases the decrease in OXBV was gradual and the decision to replace the oxygenator was made as a precautionary measure based on a combination of visual clot growth, increased resistance, and a reduced OXBV. Only the five oxygenators with a more than 30% decrease in OXBV had a sudden, sharp drop from OXBV values around 200 mL to values lower than 150 mL the next day. During these cases the oxygenators were replaced urgently to prevent further clot development and dramatic decreases in gas exchange. We have, however, no indication as to why these five oxygenators experienced the sudden drop in OXBV, as no extraordinary events were observed during prior system checks.

Even though increases in oxygenator resistance are not an accurate quantification of thrombus formation inside the oxygenator, our data showed a fair negative correlation with OXBV changes (r = -0.476; p<0.001), and a moderate negative correlation when analyzing only the replaced oxygenators (r = -0.680; p<0.001). An even more pronounced correlation was found between oxygenator resistance and an OXBV less than 187 mL (r = -0.831; p<0.001). The relationship between oxygenator resistance and clot volume thus seems to become more quantitative at higher values, hence the stronger correlation with the quantitative OXBV value. This is in line with a study by Kaesler et al. who showed that the increase in oxygenator pressure drop is modest up to a clot volume of 65 mL, and reports that pressure drop remains a vague indicator and seems to be reliable only in already advanced clotting stages [9].

Regarding all data, the oxygen transfer capacity of the oxygenators seemed to be hardly affected by increased intra-oxygenator clot formation. Only a fair, negative correlation between OXBV and O_2_ gradient (r = -0.461; p<0.001) and a fair, positive correlation between OXBV and post-oxygenator pO_2_ (r = 0.423; p<0.001) were found, whereas both O_2_ transfer (r = 0.216; p = 0.058) and O_2_ diffusing capacity (r = 0.217; p = 0.056) appeared not to be affected by decreased OXBV. When analyzing data from oxygenators prior to replacement only, or data from measurements with an OXBV less than 187 mL, the correlations between OXBV and all oxygen transfer indices were weak and/or not significant. This indicates that PLS-i oxygenators exhibiting a decreased OXBV value during the course of ECLS still have enough reserve potential to reach sufficient pO_2_ levels post-oxygenator. This observation is in contrast to other studies that stated that the incidence for an oxygenator changeout due to restricted gas transfer was up to 34% [6, 17]. In the study by Lubnow et al. one of the criteria for an oxygenator changeout was a significant decrease in the pO_2_ at the outlet of the oxygenator of more than 50% compared to the initial value. In our data this was seen in two measurements only, in two different oxygenators. The initial pO_2_ values of 437 and 423 mmHg had decreased to 157 and 192 mmHg respectively. Both oxygenators were subsequently changed out because of low OXBV values, large visible clots on the arterial side, and decreased CO_2_ transfer.

It is tempting to define an OXBV cut-off value that is indicative for an oxygenator changeout, however, all exchanged oxygenators in current study were still functioning well in terms of oxygen exchange. Therefore, it is imperative to acknowledge that the decision to changeout an oxygenator still needs consideration of multiple factors. Even though the oxygen exchange function might be preserved, intraoxygenator clots exacerbate the coagulation process and can lead to thromboembolic complications and/or unwanted systemic responses. Consequently, accurate quantification of the intraoxygenator clot development supports the decision-making for timely oxygenator changeouts.

## Conclusion

The oxygenator blood volume showed a clear decline over time during prolonged ECLS. The negative correlation with oxygenator resistance was fair in the total database, moderate in the 14 exchanged oxygenators and strong in measurements with an OXBV <187 mL. OXBV had no relevant correlations with O_2_ transfer, O_2_ diffusing capacity, O_2_ gradient and post-oxygenator pO_2_ in the total database, nor in exchanged oxygenators or measurements with an OXBV <187mL.

Nonetheless, loss of oxygenator blood volume during ECLS can aid determining intra-oxygenator clot formation, and thus in the decision for a timely oxygenator changeout.

## Supporting information

S1 Text Abbreviation list. (DOCX)
