# Supplementary material for: Dynamic oxygenator blood volume during prolonged extracorporeal life support
Source: PLoS One. 2022 Feb 2;17(2):e0263360. doi: 10.1371/journal.pone.0263360 (PMC8809600; doi:10.1371/journal.pone.0263360)
Supplement: S1 Text — (DOCX) [file pone.0263360.s001.docx]

**Dynamic oxygenator blood volume during prolonged extracorporeal life support**

Abbreviation list

Δp - Trans-oxygenator pressure gradient

aPTT - Activated partial thromboplastin time

CaO_2_ - Post-oxygenator O_2_ content

CvO_2_ - Pre-oxygenator O_2_ content

ECLS - Extracorporeal life support

ELSA - Extracorporeal life support assurance

Hb - Hemoglobin

METC - Medical review ethics committee

OXBV - Oxygenator blood volume

PaO_2_ - Post-oxygenator O_2_ tension

PgO_2_ - Sweep gas O_2_ tension

PLS - Permanent life support

PvO_2_ - Pre-oxygenator O_2_ tension

Q_p_ - Pump flow

SaO_2_ - Post-oxygenator O_2_ saturation

SvO_2_ - Pre-oxygenator O_2_ saturation

VA - Veno-arterial

VV - Veno-venous
